# Recycling of both resin and fibre from wind turbine blade waste via small molecule-assisted dissolution

**DOI:** 10.1038/s41598-023-36183-4

**Published:** 2023-06-07

**Authors:** Roksana Muzyka, Szymon Sobek, Anna Korytkowska-Wałach, Łukasz Drewniak, Marcin Sajdak

**Affiliations:** 1 Department of Air Protection, Faculty of Energy and Environmental Engineering, Silesian University of Technology in Gliwice, 44-100 Gliwice, Poland; 2 Department of Heating, Ventilation, and Dust Removal Technology, Faculty of Energy and Environmental Engineering, Silesian University of Technology in Gliwice, 44-100 Gliwice, Poland; 3 Department of Organic, Bioorganic and Biotechnological Chemistry, Faculty of Chemistry, Silesian University of Technology in Gliwice, 44-100 Gliwice, Poland; 4 Department of Optoelectronics, Faculty of Electrical Engineering, Silesian University of Technology, 2 Krzywoustego St., 44-100 Gliwice, Poland; 5 School of Chemical Engineering, University of Birmingham, Edgbaston, Birmingham, B15 2TT UK

**Keywords:** Environmental chemistry, Wind energy

## Abstract

Wind energy has significant growth potential and applicability on a global scale, but approximately 2.4% of wind turbine blades must be decommissioned annually. The majority of blade components can be recycled; however, wind blades are rarely recycled. In the present study, an alternative method was presented involving a small molecule-assisted technique based on a dynamic reaction that dissolves waste composite materials containing ester groups to recycle end-of-life wind turbine blades. This effective process requires temperatures below 200 °C, and the major component, i.e., resin, can be easily dissolved. This method can be applied to recycle composite materials, such as wind turbine blades and carbon fibre composites comprising fibres and resins. Depending on the waste, up to 100% of the resin degradation yield can be achieved. The solution used for the recycling process may be reused multiple times and can be reused to obtain resin-based components and create a closed loop for this type of material.

## Introduction

Wind is a fully renewable energy source with infinite resources and efficient technology for its utilization. Europe, China, and offshore wind turbines set new records in 2020, installing over 93 GW for a total of 742.7 GW^1^. The EU projects that new builds will raise wind energy capacity to 323 GW by 2030 from 205 GW^2^. Wind energy supplies 15% of EU electricity, and by 2030, it will supply 30%. Between 2020 and 2030, many 2000-s-era wind turbines will be directed to undergo decommissioning and dismantling^3,4^. Germany, Spain, and Denmark had 41–57% of Europe's installed wind turbines with exploitation time reaching over 15 years old in 2020^5,6^. In 2021, total power of 4 GW wind turbines (6000 turbines) may be decommissioned due to the 20-year support expiration^7^. Annually, 2.4% of all wind turbine blades in Europe are replaced^8^. Large composite materials such as wind blades are rarely recycled^9–13^, and many dismantles and landfilled blades strain the environment, resulting in a loss of chemical energy and material potential for recycling.

Wind turbine blades possess a complex composition, containing thermoplastic coatings, thermoset/glass and carbon fibre composites^14^, carbon fibre, balsa wood, and adhesives^15^. This composition makes the materials separation and further reuse of the separated fractions very difficult^16–19^. An additional 1 kW of installed wind power requires 12–15 kg of composites, including blade materials^20^. The crosslinked thermoset polymers of the outer layer composites cannot be melted or remoulded, making even the early stages of recycling problematic^21–26^. Mechanical^27–30^, thermal^31^, and chemical^32–36^ thermoset composite recycling methods have been developed by researchers. Pyrolysis and gasification thermal recycling techniques have TRL ratings of 9 and 5/6, respectively^37,38^. Unfortunately, pyrolysis conditions with temperatures exceeding 500 °C can damage fibres by retaining oxidation residues, char, or chemical structure. It is also not always economical, and its suitability is dependent on the technology used. If the thermal conversion process is to be performed as an auto-thermal process, some or all of the volatiles emitted by the process must be used. As a result, some, if not the majority, of the organic compounds recoverable from such a stream are lost. Furthermore, thermal conversion processes produce complex mixtures that necessitate additional high-temperature processes like distillation, hydrodeoxygenation, or hydrocracking before use. Solvolysis, used in this work, recovers clean, intact fibres and reuses resin, and this could close the fibre-reinforced resin composites loop^39^. Due to the high temperature (yet lower than pyrolysis or gasification) and high-pressure conditions, which allow significant volumes of solvents to be collected and reintroduced, this technique is inefficient and energy-intensive. This method offers the best cost-to-value ratio of the items despite a TRL of 5/6^26,40^.

According to a design of experiments (DOE) plan, the WTBW, GFC and CFC were solvolyses at 100–190 °C for 1–3 h under 0–60 bar of inert N_2_ in a Parr 4650 batch reactor with a 500 mL capacity chemically recycled glass, carbon fibre, and epoxy resin oligomers. All tests were carried out by the experimental design matrix of a central composite design (CCD), and the data were analysed via analysis of variance (ANOVA) to determine which factors have a statistically significant effect on the resin removal efficiency. As a catalyst, strongly basic bicyclic guanidine (TBD) was added to the process. Various amounts of TBD (0.015 or 0.025 mol ratio to fixed 1:1 mol ratio of EG: NMP) were tested to analyse their catalytic effect on the recycling process.

The main novelty presented in this paper is the identification of variables statistically significantly affecting the resin removal process from the matrix of both glass and carbon fibre-reinforced composites. Furthermore, using a design of experiments (DOE) approach, the process conditions were determined and optimised. The presented method has the significant advantage of being able to reuse the reaction mixture multiple times. Some composite waste components, particularly those derived from wind turbine waste, contain polystyrene, which can be separated by sedimentation from the reaction mixture containing ethylene glycol and n-methyl pyrrolidone. Furthermore, preliminary tests have shown that condensation in the reaction mixture can be used to separate epoxy resin precursors. In comparison to previous reports^41–43^, the presented method allows carbon and glass fibres to be recovered with greater efficiency and without additional degradation. Furthermore, monomers and dimers, which are components of the composite waste being processed, can be separated.

This is the first report to dissolve industrial epoxy elements of wind turbine blades and commercial composite debris at low temperatures and pressures. This method greatly reduces the amount of composite waste, and thermoset polymer breakdown is also revealed.

## Materials and methods

### Materials

**Figure 1:**
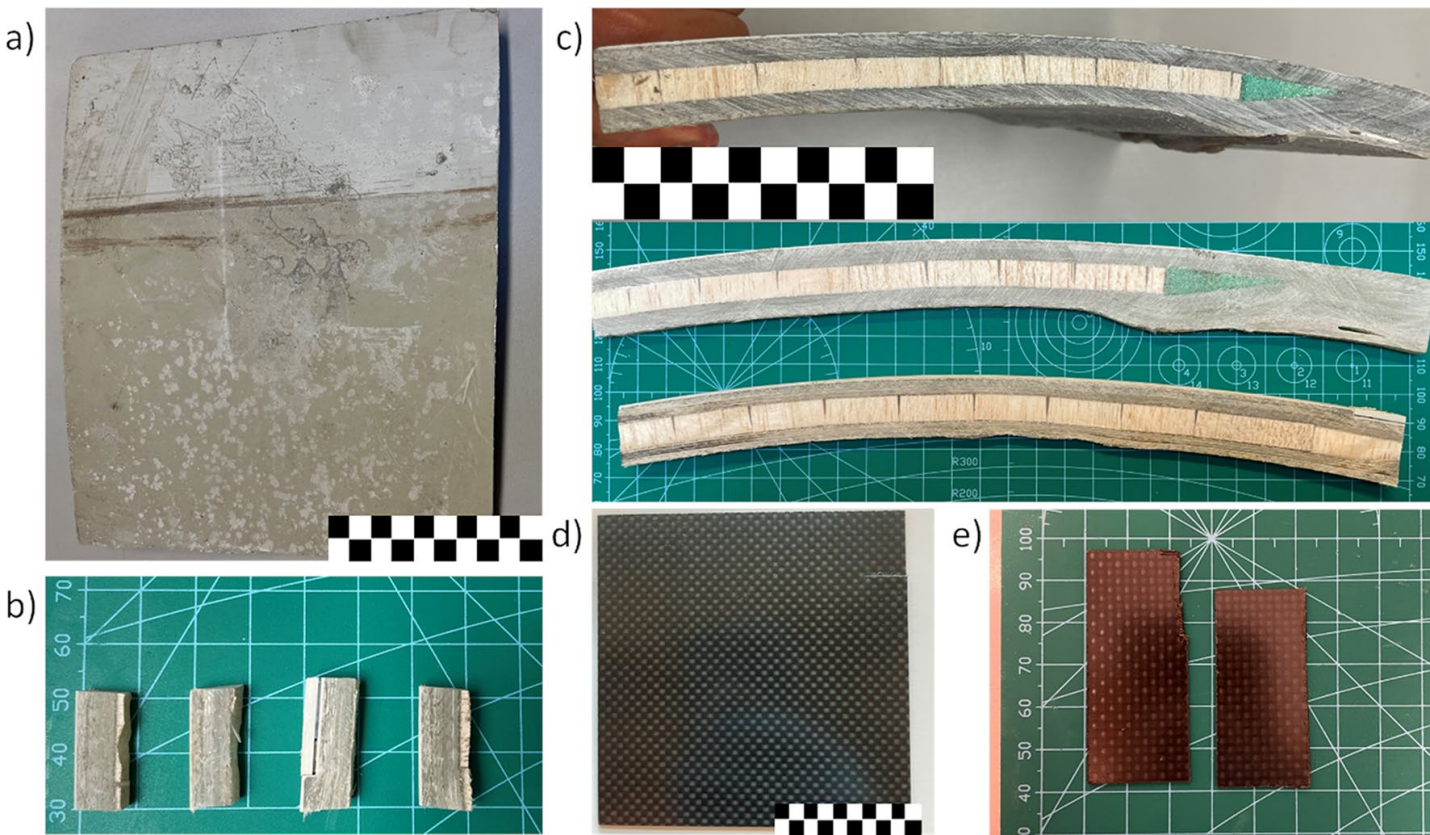
Composite waste from a wind turbine blade: (**a**) as received; (**b**) after cutting into strips; (**c**) sample for solvolysis tests; and from a carbon fibre sheet: (**d**) carbon fibre composite; (**e**) sample for solvolysis tests.

Commercial carbon fibre composite (Fig. 1d, e) and wind turbine blades (supplied as sheets with dimensions of approximately 25 × 30 cm; Fig. 1a) were used as waste samples in the solvolysis process. The samples were trimmed into thin strips (~ 0.5 cm) to capture the profile of the blade (Fig. 1b). Smaller samples (0.5 × 1 cm) were cut from the strips for solvolysis (Fig. 1c). The GFC and CFC were solvolysis as received.

The following chemicals were used in the solvolysis process and for resin synthesis from the solvolysis product stream: ethylene glycol (EG; molecular weight [MW] = 62.07 g/mol), 1-methyl-2-pyrrolidinone (NMP; MW = 99.13 g/mol), TBD (MW = 139.20 g/mol), epichlorohydrin (EPI; MW = 92.52 g/mol), isopropanol (i-Pr), sodium hydroxide (NaOH), acetic acid, propylene carbonate (PC; MW = 102.09 g/mol), and glycerol triacetate (TAG; MW = 218.21 g/mol). All reagents were purchased from Sigma‒Aldrich (St. Louis, MO, USA) and used as received.

### Analytical methods

Test samples were analysed using the Fourier transform infrared spectroscopy (FTIR) by Thermo Nicolet, model IS50. Sample spectra were recorded with the step size was 0.05/cm, and the range was 400–4000/cm. All measurements were carried out at room temperature, and a total of 64 scans were acquired for each sample under consideration. The spectral processing was carried out with the help of OMNIC version 9.

In addition, to better understand the composition of the resin matrix, samples were subjected to analytical pyrolysis by Pyrolyzer EGA/PY-3030D Multi-Shot Pyrolyzer for Py-GC/MS analysis (Frontier Laboratories Ltd, Fukushima Japan). The pyrolysis temperature was set at 500 °C, while the GC oven temperature was gradually raised from 45 to 275 °C at a rate of 5 degrees Celsius per minute. A portion of the sample vapours generated in the furnace was divided (at a ratio of 1/50), and a portion was sent to the column at a flow rate of 1.91 mL/min and a pressure of 27.3 kPa, while the remainder was vented. The vapours were separated using a Shimadzu QP-2010 Ultra Plus (Japan) gas chromatogram with a temperature-programmed capillary column and analyzed at 70 eV using a Shimadzu MS-QP2010SE mass spectrometer. The Zebron ZB-5 capillary column from Phenomenex was used (with a 5 percent diphenyl and a 95% dimethylpolysiloxane stationary phase, a column length of 30 m, a column ID of 0.32 mm, and a thickness of 0.10 m). The mass spectrometer should be configured to the following settings: ion source heater 250 °C, interface temperature 300 °C, vacuum 10–5 Pa, m/z range 45–300, and scan speed 1428. Shimadzu (NIST17.0) post-run software was utilized to further examine each experiment's chromatograms and spectra.

Furthermore studied samples were subjected to NMR analyses: soluble fractions, separated during extraction with chloroform in a Soxhlet apparatus; the products of the solvolysis process and the condensation product of one of the latter with epichlorohydrin. 1H NMR and 13C NMR spectra were recorded at 25 °C with the aid of a 600 MHz Varian spectrometer in CDCl_3_ as a solvent. Tetramethylsilane (TMS) was used as an internal reference.

### Recycling of glass, carbon fibre, and epoxy resin oligomers

Chemical recycling was performed at 100–190 °C for 60–180 min under 30–60 bar of an inert N_2_ atmosphere in a 500 mL Parr 4650 batch reactor with the transesterification catalyst, TBD, ethylene glycol and 1-methyl-2-pyrrolidinone in a 1:1 mol ratio. Under each set of solvolysis conditions, 1 g of sample and 20 mL of solution were used. To avoid overheating, the Parr 4650 batch reactor was calibrated before each test. After each experiment, the sample was filtered to extract fibre residues. After filtration, the liquid sample was analysed by nuclear magnetic resonance spectroscopy (NMR), and the solid residue on the filter was washed, dried, and weighed. After weighing the fibre sample content, it was burned to analyse the contents of the ash. The solvolysis process resin degradation yield (RDY) was determined using Eq. (1),

1 $$RDY=\frac{{w}_{1}-{w}_{2}}{{w}_{0}}$$

where w_1_ is the sample weight before solvolysis, w_2_ is the sample weight after solvolysis, and w_0_ is the epoxy resin weight before solvolysis^42^.

After process condition optimization, the solvolysis was scaled up to a 2 L unpressurized batch reactor with a reflux condenser and a nitrogen supply. The liquid sample after solvolysis was subjected to an addition reaction to investigate the potential for resin recovery from the composite materials. Specifically, 50 mL of liquid solvolysis products, 20.0 mL of EPI, and 10.0 mL of i-Pr were added to a 100 mL triple-neck flask equipped with a reflux condenser, thermometer, and dropper for the addition reaction. To ensure complete mixing, the reaction mixture was heated at 50 °C for 5 min. The addition reaction was allowed to proceed for 15 min at 67–70 °C by adding 1.5 mL of NaOH (0.15 mol). After cooling to 55 °C, 15.5 mL of NaOH was added to the reaction mixture, and condensation occurred for 90 min. After complete condensation, water was added to the mixture, which was then heated to 80 °C for 10 min to dissolve the generated sodium chloride. After 30 min, the reaction mixture was added to a separatory funnel to separate the aqueous phase from the organic phase.

### Recycling mechanism

**Figure 2:**
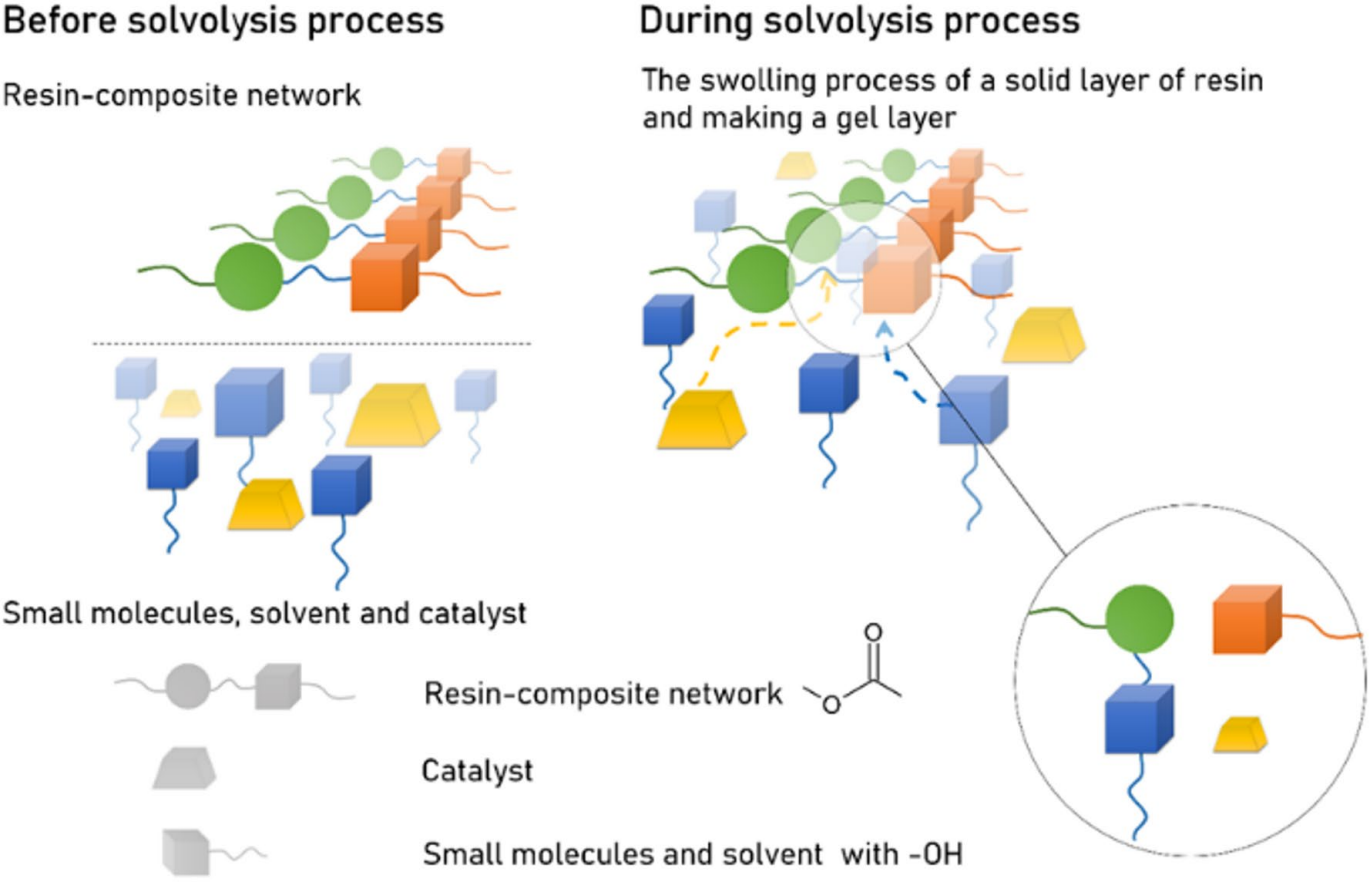
The solvolysis process involves a bond exchange reaction between hydroxy groups in the solvent and ester bonds in the resin-composite network.

The degradation of the resin-composite network is a crucial step in the recycling of waste composite materials. Figure 2 shows the mechanism of the small molecule-assisted dissolution approach. The recycling solvent comprises EG and organic solvent (i.e., NMP) in a molar ratio of 1:1, along with various amounts of the transesterification catalyst (i.e., TBD) ranging from 0.015 to 0.025 mol. The transesterification-type bond exchange process occurs between hydroxy groups in the solvent and ester groups in the resin-composite material. EG cleaves ester bonds in the polymer network, causing the thermoset substrate to dissolve into oligomers and monomers. A surface layer model with three layers (solid swelling layer, gel layer, and pure polymer layer) was used to characterize the thermosetting polymer dissolution kinetics (Fig. 2)^44^. The diffusion of the organic solvent (i.e., NMP) accelerates the swelling of the thermosetting resin, which allows significant amounts of EG and TBD to enter the polymer network. A thicker gel layer forms at the surface layer, where the bond exchange reaction occurs, and the resin surface gradually erodes.

### Recyclability of resin-based waste composite

**Figure 3:**
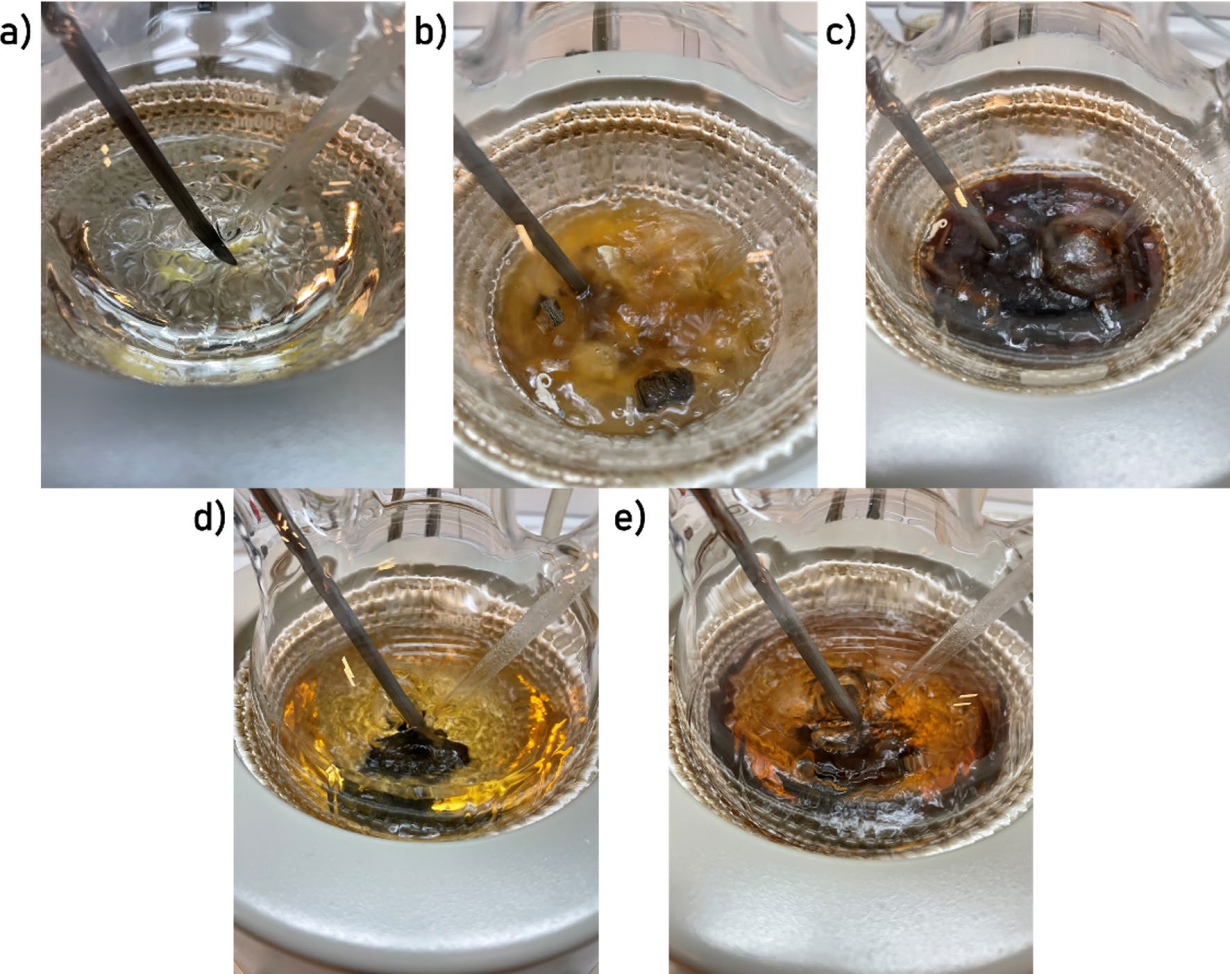
Solvolysis solution during the tests (**a**) without sample, (**b**) with turbine blade sample after 1 h, (**c**) with turbine blade sample after 3 h, (**d**) with carbon-fibre composite after 1 h, (**e**) with carbon-fibre composite after 3 h,

The small molecule-assisted waste composite recycling method includes two basic steps: (1) the recycling solution dissolves the resin of the waste composite, and (2) the carbon or glass fibre-based waste composite is removed from the products. In the first step, the solution changes from transparent to yellow and dark orange following the reaction between the transesterification catalyst, resin, and other chemicals present in the samples as present in Fig. 3.

A total of 29 solvolysis experiments were carried out under various processing conditions, according to the experimental design, i.e., using a temperature range of 100–190 °C, a pressure range of 0–60 bar, a duration range of 1–3 h, and a TBD catalyst concentration range of 0.015–0.025 mol. Owing to the deep penetration of small molecules, the thermosetting polymer substrate eventually deteriorates. At high temperatures, polymers with low melting points were also dissolved. After the dissolution process, the sample was filtered to separate the liquid from the glass fibres, which were then washed with isopropanol, dried at 105 °C, and weighed.

**Figure 4:**
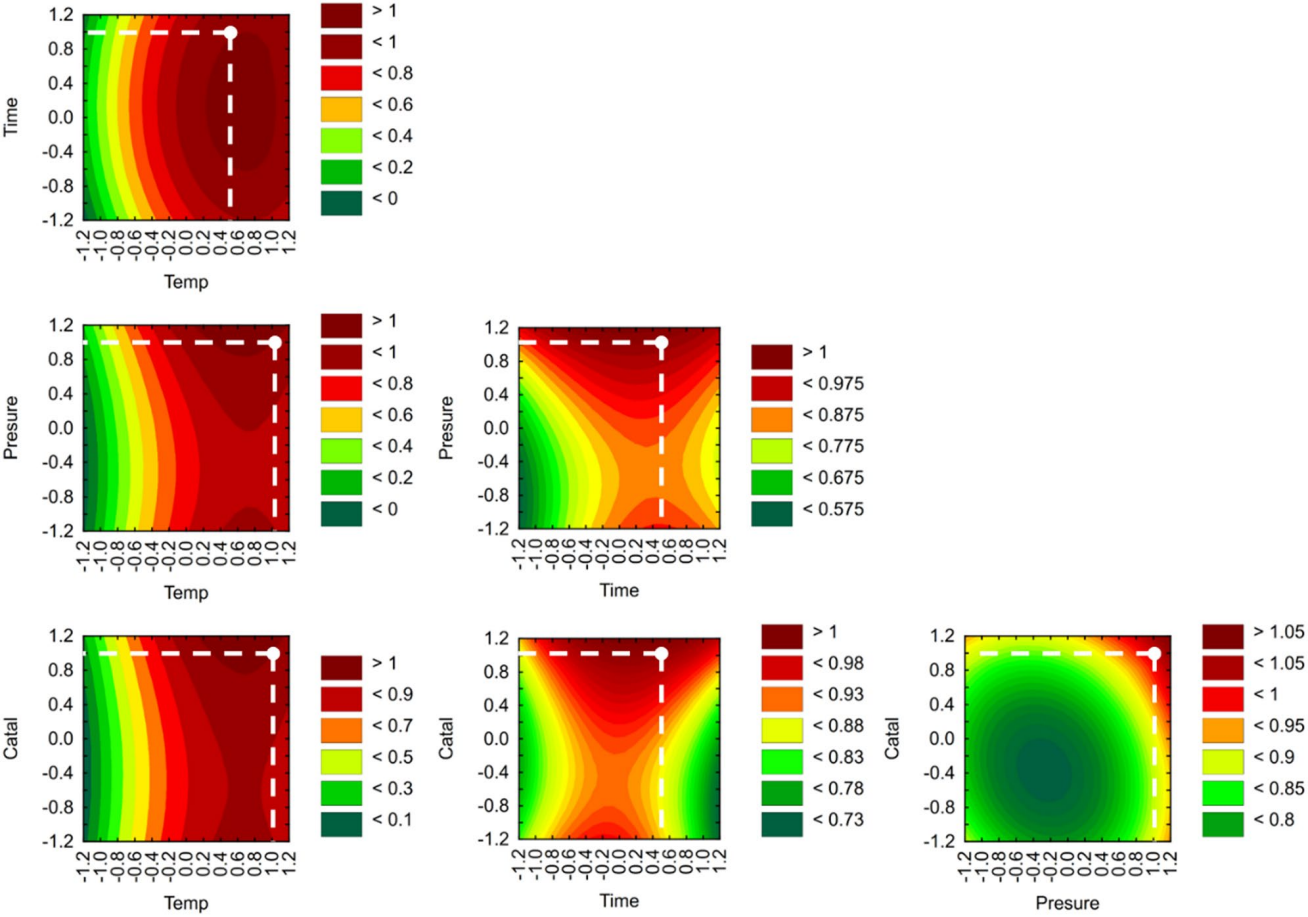
Response surface of the utility function, assuming a maximized RDY.

Based on the results from these 29 experiments under various conditions, the optimal conditions in terms of maximizing the resin removal from the WTB composite samples are as follows: processing temperature = 190 °C, duration = 2.5–3 h, pressure = 60 bar, and TBD content = 0.025 mol (Fig. 4). The conducted analysis showed small, statistically significant effects of the solvolysis duration and catalyst concentration (alpha confidence interval = 0.05) on the standard deviation of the RDY value; this relationship is related to the variable composition of the tested WTB samples. Specifically, this variability is due to the inconsistent amount of glass fibre used in the composite construction.

The experimental plan and ANOVA method revealed the statistically significant variables that affect the solvolysis process in this study. We also evaluated the interactions between the selected variables and determined whether they had statistically significant effects on the degree of resin removal from WTB samples. The optimized processing conditions led to resin removal yields ranging from 4.36 to 34.43%. The factors with the most statistically significant effect on the resin removal yield were temperature, pressure, and time. ANOVA confirmed that there was a negative correlation between time and pressure and a positive correlation between the reaction time and the amount of catalyst. The negative impact of the interaction between processing time and increasing pressure on the RDY could be avoided by conducting the process at atmospheric pressure. Under these conditions, there is a local maximum for the RDY value, and the interaction between the processing time and increasing pressure does not negatively impact the processing efficiency.

**Figure 5:**
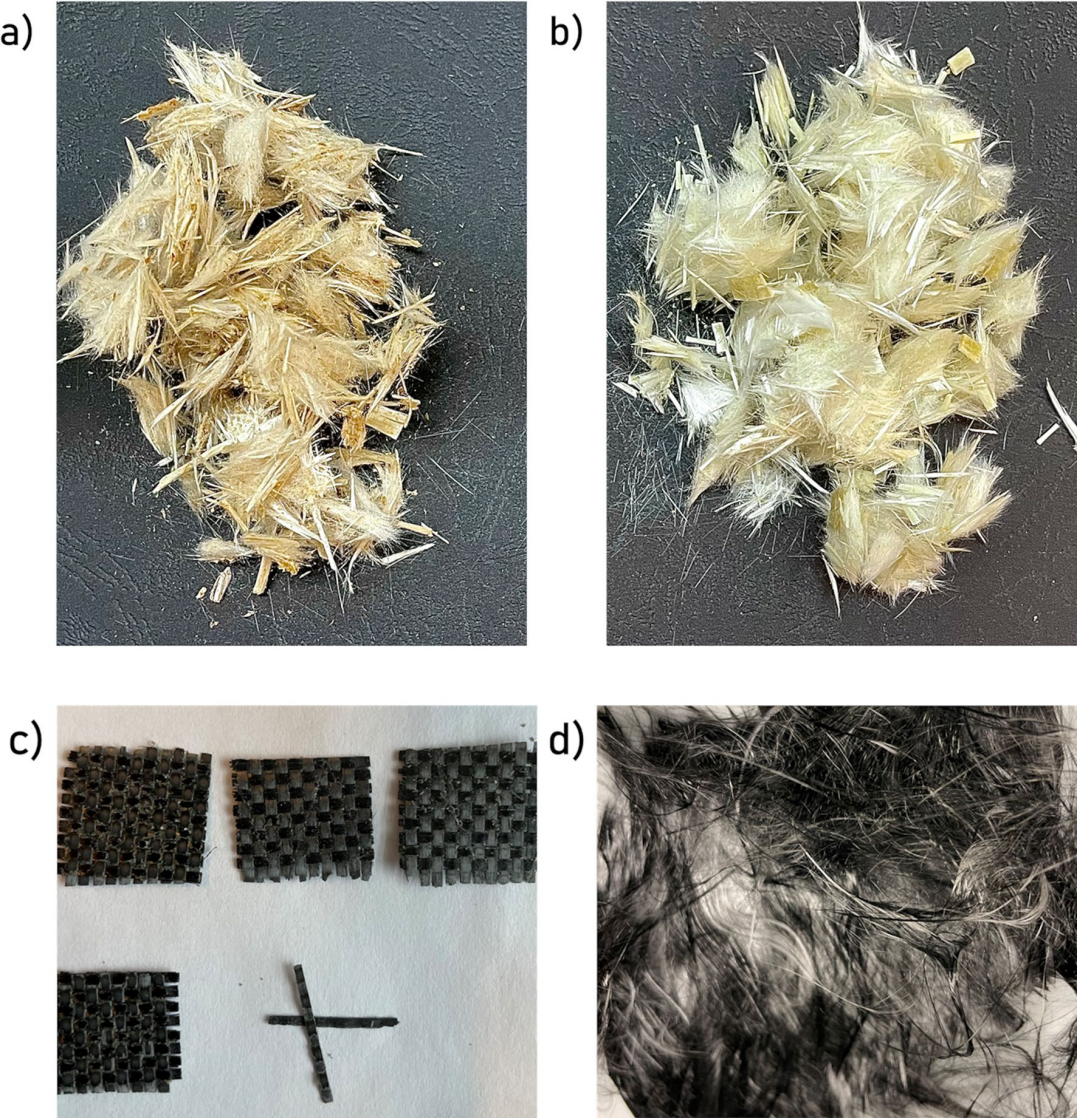
Glass fibre obtained during the solvolysis process under optimal conditions (**a**) after one step and (**b**) after a second step, carbon fibre obtained during the solvolysis process under optimal conditions on the 2 L scale: (**c**) after 3 h, (**d**) after 6 h.

After the optimization of solvolysis for WTBW the cascade approach was applied. In the WTBW case, the cascade process more than doubled the resin removal rate from the material. Additionally, after the second step, the overall amount of RDY resin that was removed reached 80% (Fig. 5b). Comparable results were obtained for the CFC using waste from WTBW that had been subjected to the described process only once (32.5%; Fig. 5a).

After that, the solvolysis of commercial carbon fibre composites was then scaled up to 2 L and run out of an unpressurized batch reactor. At this time, the solvolysis process was carried out in the same optimal conditions and at twice the time—up to 6 h. After that, very good results, close to 100% resin degradation yield, were obtained. These results are presented in Fig. 5c and d. After washing, very clear fibres were obtained.

**Figure 6:**
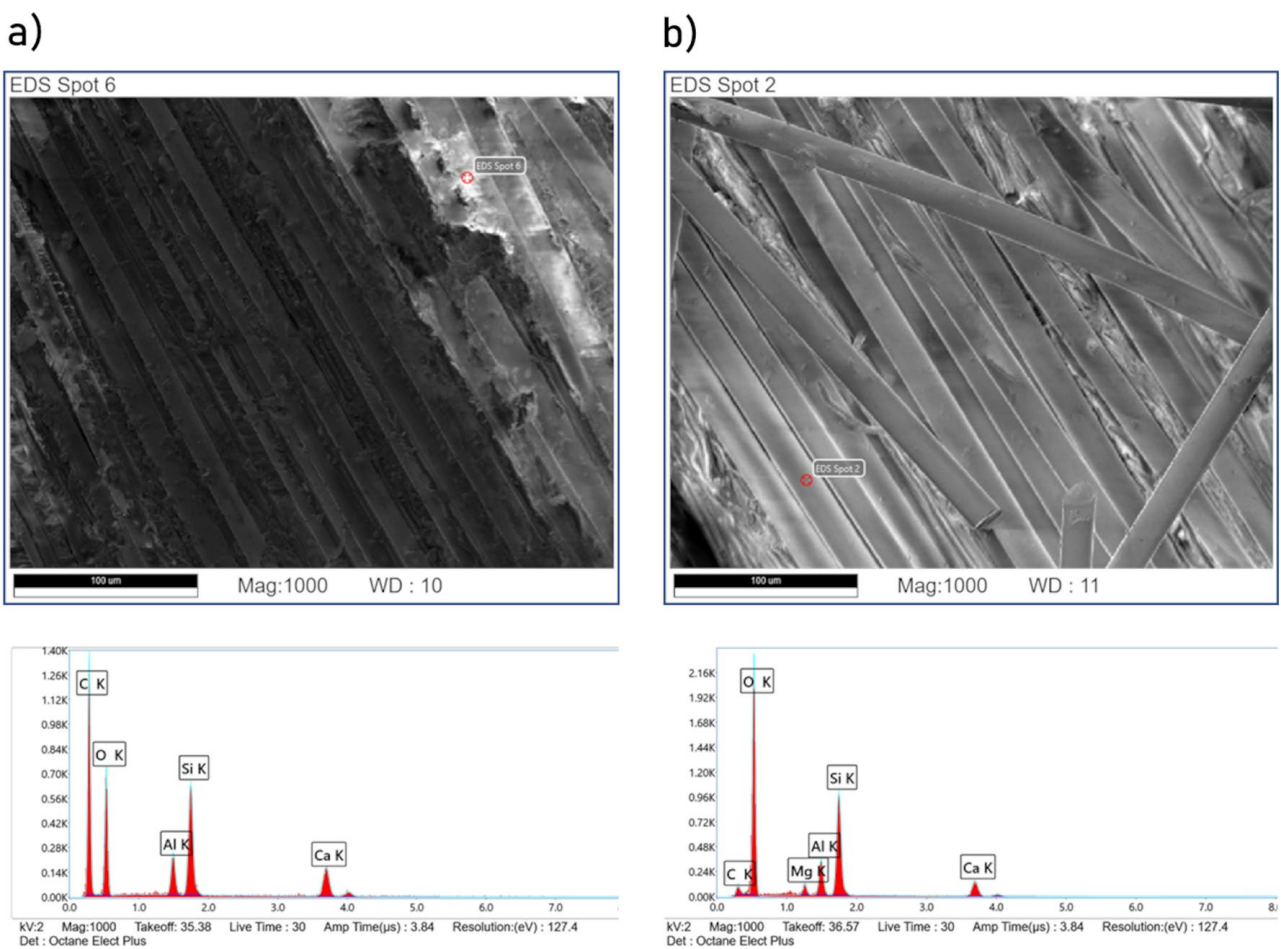
SEM–EDS images of (**a**) glass fibre composite, (**b**) glass fibre after washing and unification.

It could be seen that after washing, very clear fibres were obtained, which was also confirmed by SEM–EDS analysis (Fig. 6). SEM–EDS clearly show the smooth surface of larger-scale fibres.

Based on Py-GC–MS as well as NMR analysis, it was found that in both cases the composite matrices contained a crosslinked and uncrosslinked fraction of epoxy resin, 1,2-benzene dicarboxylic acid (phthalic acid) and phthalic acid derivatives, and in the case of waste from wind turbine blade also styrene and polystyrene. The main compounds identified in the pyrogram from wind turbine blades were: styrene (3.05 min), 1,2-benzene dicarboxylic acid (12.79 min), cyclopropyl phenylmethane (21.97 min), 3-(2-Phenylethyl)benzonitrile (35.07 min) 1-docosene (46.45 min) and heptacosane (49.44 min). The results of the analytical pyrolysis of studied composites correspond to epoxy resin and unsaturated polyester resin^45–47^. The ATR-FTIR spectra indicate that at high wavenumbers, the FTIR spectrum contains an absorption band at 3400 cm^-1^ attributable to the O–H stretching mode of hydroxyl groups, showing the existence of dimers or high molecular weight species. Bands at 3060/cm correspond to the C–H stretching vibration mode of the epoxide group, while those at 2960 and 2872/cm relate to the –CH_2_ and –CH_3_ stretching vibration modes of aromatic and aliphatic chains, respectively. Bands at 1722/cm are assigned to the C=O stretching mode. Some weak intensity bands at 1600, 1493, 1452, and 1374/cm indicate the presence of N–H functional groups of amines, as well as C–N bonds of amine and imide groups presumably belonging to the catalyst and modifier added to the modified epoxy resin^48^. Furthermore, the presence of bands at 1232, 1153, and 1063/cm confirms the existence of C–O–C and C–O stretching vibrations due to ether linkage. The epoxide groups in the resin are identified by the characteristic absorption bands cantered at 984 and 908/cm, which are connected with the glycidyl ether functionality and the C–O stretching vibration mode of the oxirane ring, respectively. Finally, bands at 742/cm were assigned to C–H out-of-plane absorptions of substituted aromatic rings.

After liquefaction, the liquid products were analysed via nuclear magnetic resonance to ascertain their viability for further application. The NMR analyses were performed on the following samples: soluble fractions of WTB, which were separated during extraction with chloroform in a Soxhlet apparatus; products of WTB and condensation products of WTB solvolysis with EPI. ^1^H- and ^13^C-NMR spectra at 25 °C were recorded on a 600 MHz Varian spectrometer using CDCl_3_ as the solvent and tetramethyl silane (TMS) as an internal reference. ^1^H-NMR examinations of the soluble portion of wind turbine blade samples revealed the presence of styrene, 1,2-benzenedicarboxylic acid (phthalic acid), and phthalic acid derivatives (Supplementary materials S 1a). Additionally, the uncross-linked fraction of epoxy resin was detected. In the liquid samples obtained after the recycling process (Supplementary materials Sb, c), monomers and dimers were detected after the transesterification process. Bisphenol A, which is a substrate used in the production of epoxy resins, is one of the most important compounds identified in the analyzed samples.

It is important to note that the manufacturing technology for wind turbine blades has undergone changes over time, resulting in alterations to the composition, quantity, and variety of epoxy resins and additives utilised. But, the epoxy resins as a group have remained unaltered, as can be asserted.

**Figure 7:**
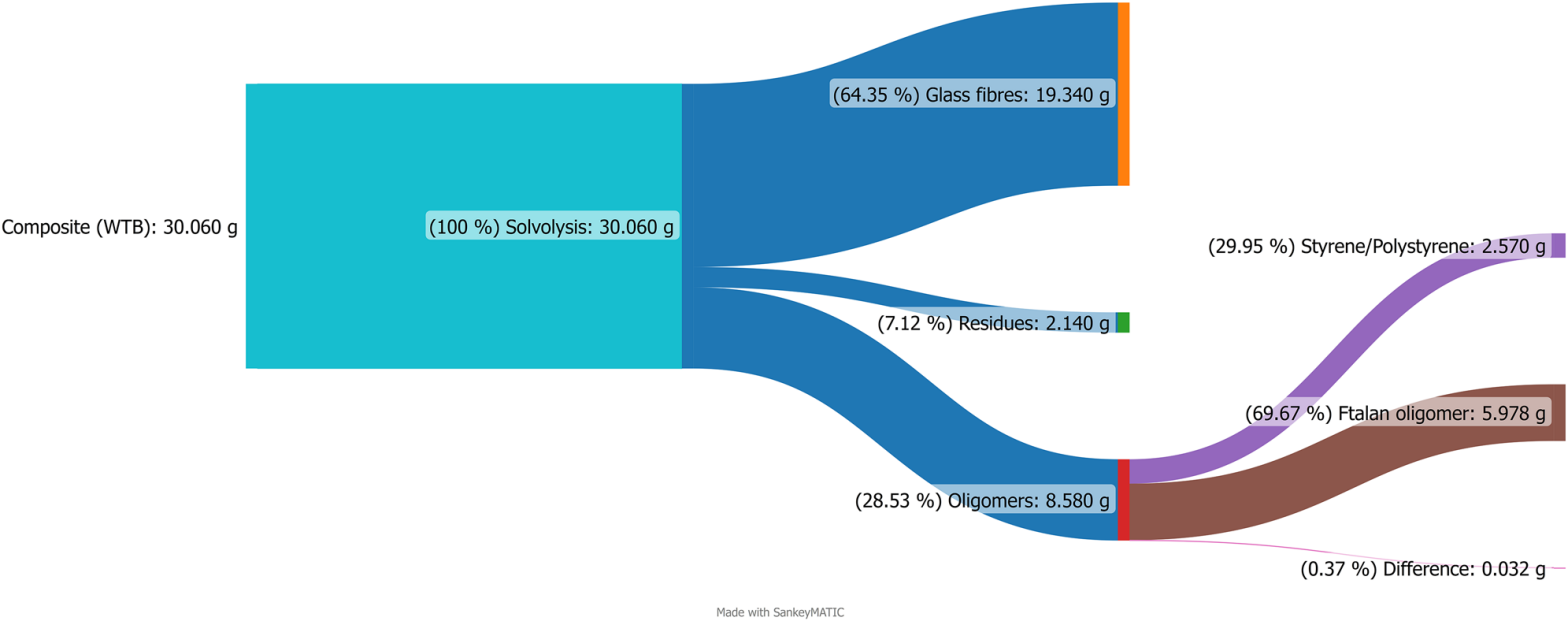
Sankey diagram for solvolysis process of composite (excluding the mass of solvents).

Based on the chemical analyses, it was calculated that during the liquefaction process under optimised conditions 80.04% of the polymer matrix, i.e., 8.58 g, was removed from the 30.06 g composite waste sample containing 64.35% of the fibres (19.34 g). On the fibre surface, 2.14 g of undissolved polymer matrix remained. The NMR analysis allowed determine that the post-liquefaction sample contained an unsaturated polyester resin comprising bis(2-ethylhexyl) phthalate and maleic anhydride (on the basis of Py-GC–MS and NMR analysis), which, when converted into an oligomer, was cured with styrene in an amount of 30–40 wt.%. This amount was confirmed by Py-GC–MS analysis. This analysis yielded 39.46% styrene in the pyrolysis products of the composite material. Due to the fact that the samples tested were real waste and no exact information was available on the raw materials used for the production of the wind turbine blades, it was assumed in the results presented below that 30 wt.% styrene could have been used to cure the unsaturated polyester resin, and not nearly 40 wt.% as shown by the Py-GC–MS analysis. This is because, during pyrolysis, some of the styrene may additionally come from phthalate decomposition. In addition, NMR analysis showed that the molar ratio of the phthalate-containing oligomer to NMP is 0.0054. Assuming an average oligomer mass of 1107 g/mol, it was calculated that there is almost 6 g of oligomer (5.978 g) in the liquid product, which makes it possible to close the mass balance presented in Fig. 7 quite satisfactorily. It can therefore be concluded that the recovery efficiency for the oligomers (phthalate oligomer, styrene, and polystyrene) from the polymer matrix in the case studied was approximately 80%.

The method described above allows the isolation of 19.34 g of glass fibre with a yield of 90%, while the residual 2.14 g, equivalent to 10%, represents the resin that is incapable of dissolving in the composite matrix under the experimental conditions employed. Moreover, the technique allowed the isolation of 8.58 g of oligomers, corresponding to an 80% weight yield of the polymer matrix in the experimental residue (excluding glass fibre). The oligomer composition comprises of styrene/polystyrene and phthalate oligomers, constituting approximately 30% and 70% of the total mixture, respectively.

### Synthesis of resin precursors from liquid solvolysis products via condensation

The presence of bisphenol An in the liquid solvolysis products made it possible to test the hypothesis that a resin could be obtained by condensing bisphenol A with EPI without first purifying the reaction mixture. Obtaining a resin from such an intermediate would demonstrate that resins used in industry can be recovered and reused. The condensation of bisphenol A from the solid products after solvolysis in the presence of EPI indicated that polycondensation could produce an epoxy resin transition product known as bisphenol A propoxate. The NMR spectrum of the solid condensation products contains signals attributed to polystyrene. Polystyrene was most likely produced through the polymerization of styrene (which was present in the WTB samples) under the applied solvolysis conditions.

### Comparison with other methods

There are ways in the literature that belong to chemical recycling procedures that use different low-molecular-weight compounds as potential solvents than the one used in this case. Acetone, low-molecular-weight alcohols such as ethyl alcohol or 2-propanol, acetic acid, ethylene glycol (EG), propylene glycol (PG), n-methyl pyrrolidone (NMP), propylene carbonate (PC) and glycerol triacetate (TAG) are examples of these molecules. To suggest a non-pressurised technique that could be employed more broadly and securely on a bigger scale than subcritical or supercritical systems, low-boiling solvents were omitted from the comparisons, and only high-boiling solvents were evaluated. The comparison also included propylene carbonate, a relatively safe and frequently used solvent in the polymer industry. For comparison, a 1 g sample of wind turbine blade waste was employed, and solvolysis operations were carried out under reproducible process conditions: 190 °C temperature, 3 h process time, with and without the presence of a catalyst. The results of the comparative analyses are shown in Table 1.

**Table 1:** The results of the comparative analyses of the solvolysis test with various types of solvents.

Solvent [mol]	Catalyst TBD [mol]	Resin degradation yield (RDY) [%]
TAG/PC (1:1)	0.01	7.46
EG	0	0.20
Acetic acid	0	0.31
EG/NMP	0	11.53
EG/PC	0.01	3.51
EG/PC*	0.01	30.10
EG/NMP	0.025	36.41
EG/NMP—two-stage process—cascade	0.025	80.04
EG/NMP (6 h for carbon fibre composite)	0.025	98.6

When utilising an identical molar ratio of ethyl glycol and propylene carbonate, it was discovered that the resulting mixture, even when heated, is rather dense and severely impedes proper catalyst dissolving (TBD). Forced stirring (EG/PC*) of the entire mixture was required in this case to achieve an acceptable result. It has been observed that in the case of wind turbine blade waste, the cross-linked resins utilised appear to be too stable to be degraded using merely low-molecular-weight compounds, as can be shown with some of the solvents successfully used by another researcher^41–43^. The process times of up to 16 h stated in the literature may be a severe impediment to the feasibility of the recycling process. Furthermore, the literature^42^ does not indicate under what pressure the processes were carried out in some cases, as the study used both ethylene glycol and propylene glycol, which have boiling points of 197 °C and 188.2 °C, respectively, so chemical recycling using them for 16 h at 270 °C had to be carried out under pressure in a suitably adapted reactor.

## Conclusions

This study proposes a flexible reaction approach for recycling waste wind turbine blades that is effective and environmentally friendly. At temperatures below 200 °C, a transesterification reaction efficiently dissolved thermoset ester-containing resins in waste wind turbine blades and carbon fibre composites. The glass and carbon fibre materials that were obtained were easily separated from the product solution. Experiment results showed that this method could recycle a wide range of genuine carbon and glass fibre resin composites with high efficiency (almost 100%), including wind turbine blades made from epoxy-anhydride and polyester resin substrates. Because the concept described herein can be applied to other types of ester-containing substrate polymers, incorporating ester groups into the substrate polymer network could be a promising strategy for environmentally friendly recycling. Importantly, after processing, the liquid fraction can be used to separate monomers from epoxy resins, such as styrene and epoxy resin precursors.

To the best of our knowledge, this is the first time this approach has been used to process a complex real-world matrix of this type with a complex DOE approach. Once fully optimized, this technology could be used to recover the glass fibres that comprise the composite or to produce epoxy resin precursors for use elsewhere. Our study showed that in the case of WTB composite which was cross-linked by styrene, without stabilization styrene self-polymerises in the post-processing mixture, which facilitates its removal. The post-processing mixture can be recycled until it is saturated with the leached oligomer and then regenerated by recovering the oligomers.

Based on the findings presented here, future research should concentrate on further optimizing the solvolysis process, such as by using a specific solvent mixture based on the composition of the composite, using other transesterification catalysts, or changing the technical sequence. The developed approach is also compared to previously reported strategies for chemically recycling waste-stained glass turbine blades in this paper. In terms of reducing the amount of waste produced by wind turbine blades and carbon fibre composites, the strategy used in this study was the most successful. Scientists will continue to look for other possible catalysts that will speed up the breakdown of the epoxy resin-composite matrix as research on this topic progresses. Furthermore, further research will focus on determining the commercial feasibility of the presented solution depending on the type of waste materials.

## Supplementary Information

Supplementary Figures.

## Data Availability

The datasets generated and analysed during the current study are available in the Mendeley Data repository^49^, “Chemical recycling of End-of-Life wind turbine blades”, V1, https://doi.org/10.17632/7hjmb2bxdh.1, https://data.mendeley.com/datasets/7hjmb2bxdh/1.

## Abbreviations

WTBW: Wind turbine blade waste
GFC: Glass fibre composites
CFC: Carbon fibre composites
EG: Ethylene glycol
NMP: 1-Methyl-2-pyrrolidinone
TBD: 1,5,7-Triazabicyclo[4.4.0]dec-5-ene
EPI: Epichlorohydrin
i-Pr: Isopropanol
NaOH: Sodium hydroxide
NMR: Nuclear magnetic resonance
