# Supplementary material for: Recycling of both resin and fibre from wind turbine blade waste via small molecule-assisted dissolution
Source: Sci Rep. 2023 Jun 7;13:9270. doi: 10.1038/s41598-023-36183-4 (PMC10247820; doi:10.1038/s41598-023-36183-4)
Supplement: Supplementary file 1 — Supplementary Figures. [file 41598_2023_36183_MOESM1_ESM.docx]

**Supplementary materials**

Recycling of both Resin and Fibre from Wind Turbine Blade Waste via Small Molecule-Assisted Dissolution

Roksana Muzyka^1^, Szymon Sobek^2^, Anna Korytkowska-Wałach^3^, Łukasz Drewniak^4^ & Marcin Sajdak^1,5,*^

^1^ Department of Air Protection, Faculty of Energy and Environmental Engineering, Silesian University of Technology in Gliwice, 44-100 Gliwice, Poland

^2^ Department of Heating, Ventilation, and Dust Removal Technology, Faculty of Energy and Environmental Engineering, Silesian University of Technology in Gliwice, 44-100 Gliwice, Poland

^3^ Department of Organic, Bioorganic and Biotechnological Chemistry, Faculty of Chemistry, Silesian University of Technology in Gliwice, 44-100 Gliwice, Poland

^4^ Department of Optoelectronics, Faculty of Electrical Engineering, Silesian University of Technology, 2 Krzywoustego St., 44-100 Gliwice, Poland

^5^ School of Chemical Engineering, University of Birmingham, Edgbaston, B15 2TT, Birmingham, United Kingdom

* Corresponding author. Email: [Marcin.Sajdak@polsl.pl](mailto:Marcin.Sajdak@polsl.pl)


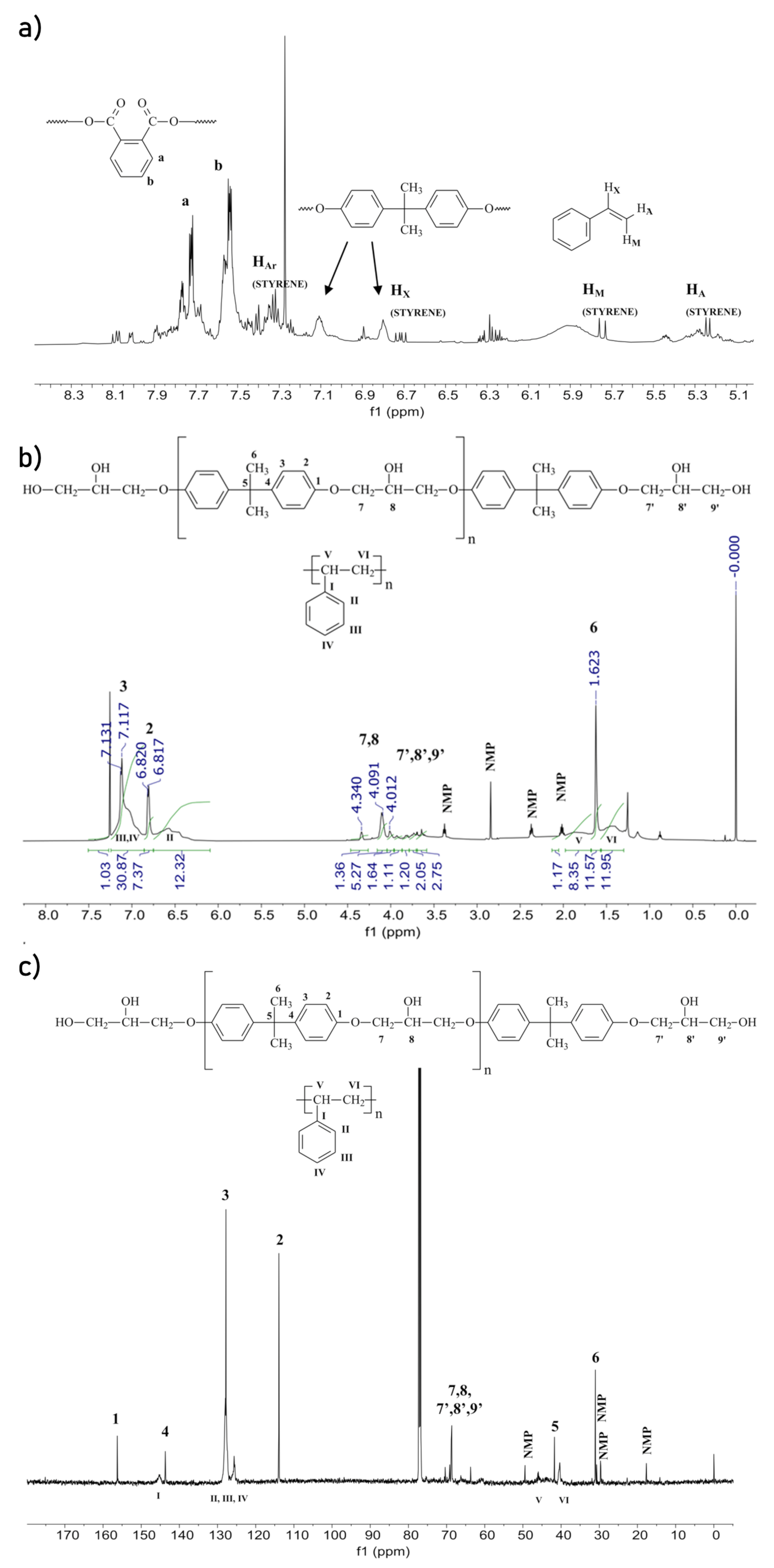


**Figure S1.** Aromatic and conjugated double-bond protons in the ^1^H-NMR spectrum of a WTB chloroform extract


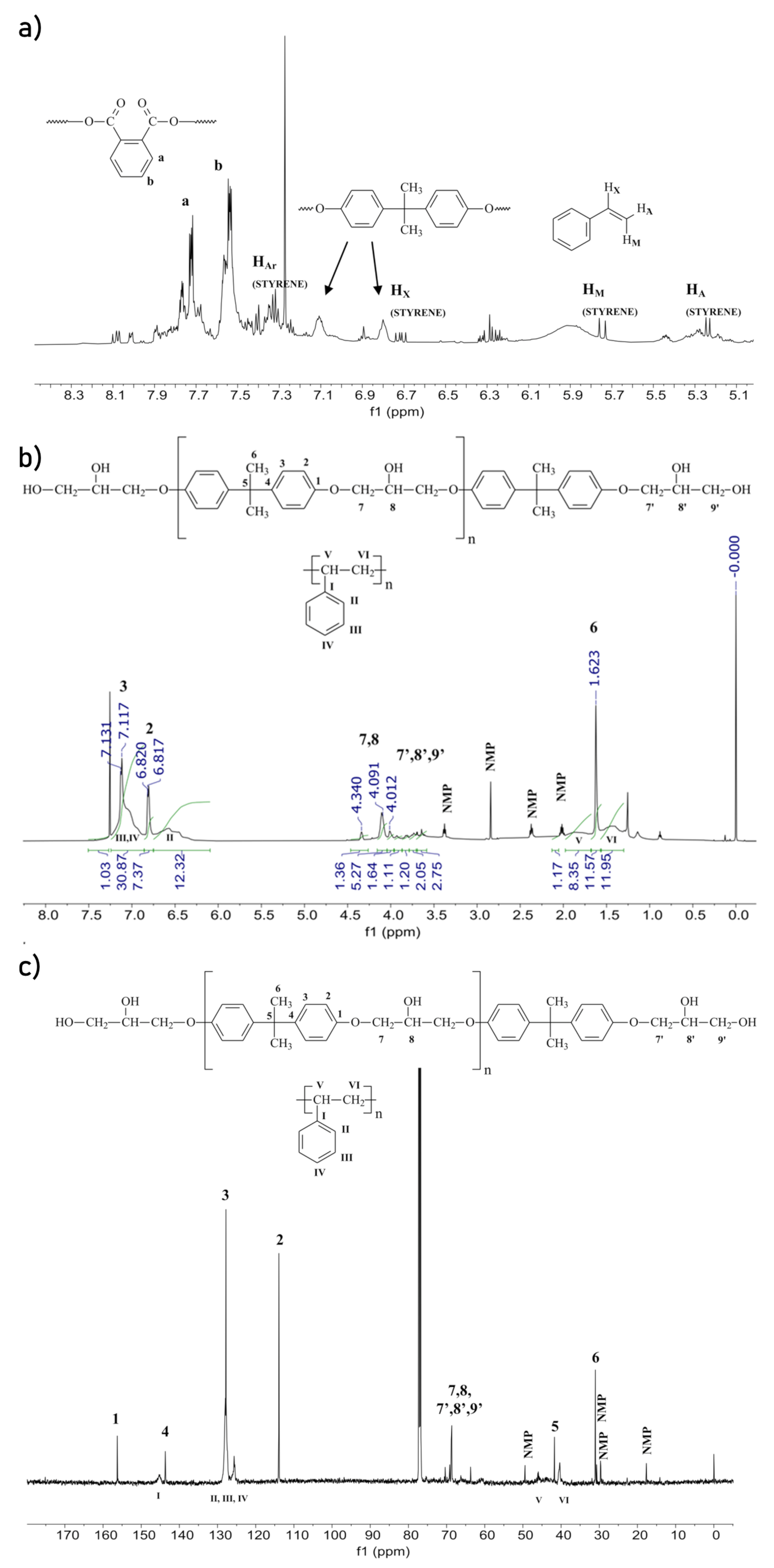


**Figure S2** The ^1^H-NMR spectrum of the solid solvolysis products obtained under the optimal conditions after condensation with epichlorohydrin, (c) ^13^C-NMR spectrum of solid solvolysis products obtained under the optimal conditions after condensation with epichlorohydrin.


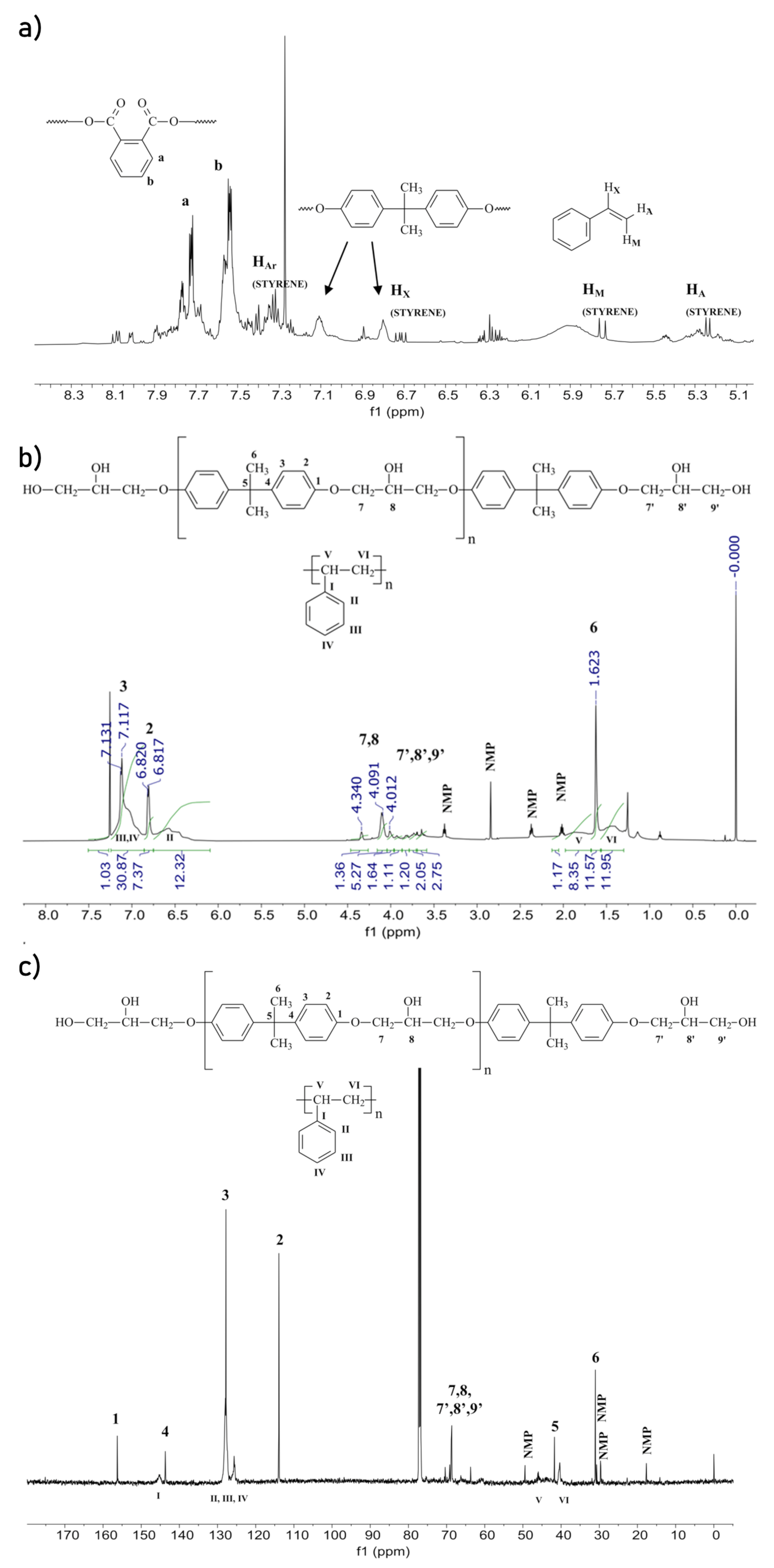


**Figure S3.** The ^13^C-NMR spectrum of solid solvolysis products was obtained under optimal conditions after condensation with epichlorohydrin.
